# Up-regulation of REG3A in colorectal cancer cells confers proliferation and correlates with colorectal cancer risk

**DOI:** 10.18632/oncotarget.6473

**Published:** 2015-12-04

**Authors:** Ying Ye, Ling Xiao, Su-Juan Wang, Wei Yue, Qiao-Shan Yin, Meng-Yao Sun, Wei Xia, Zhi-Yi Shao, Hong Zhang

**Affiliations:** ^1^ Central Laboratory, Shanghai Seventh People's Hospital, Shanghai, PR China; ^2^ Department of Pharmaceutical Botany, School of Pharmacy, Second Military Medical University, Shanghai, PR China; ^3^ Department of Nuclear Medicine, Shanghai Seventh People's Hospital, Shanghai, PR China

**Keywords:** colorectal cancer, REG3A, AKT, ERK1/2

## Abstract

Colorectal cancer (CRC) is one of the most common malignancies in the world. Previous studies have investigated the altered expression of regenerating islet-derived 3 alpha (REG3A) in various cancers. We aimed at exploring the biological function and the underlying molecular mechanism of REG3A in CRC. In this study, REG3A was found elevated in CRC compared with normal tissues. Further, high REG3A expression level was correlated with bigger tumor size, poorer differentiation, higher tumor stage and lower survival rate. Knockdown of REG3A in two CRC cell lines, LOVO and RKO, significantly inhibited cell proliferation, and increased cells population in G1 phase and cell apoptotic rate. We also found that down-regulation of REG3A in CRC cells notably inhibited cell migration and invasion. Gene set enrichment analysis on The Cancer Genome Atlas dataset showed that Kyoto Encyclopedia of Genes and Genomes (KEGG) DNA replication and base excision repair (BER) pathways were correlative with the REG3A expression, which was further confirmed in CRC cells by Western blot. Moreover, we confirmed the interaction of REG3A and fibronectin in CRC cells. We also found that there was a positive correlation between REG3A expression level and the AKT and ERK1/2 phosphorylation status. These collective data indicated that REG3A overexpression promotes CRC tumorigenesis by activating AKT and ERK1/2 pathways. REG3A may serve as a promising therapeutic strategy for CRC.

## INTRODUCTION

Colorectal cancer (CRC) is the third most commonly diagnosed tumor type in males and the second in females in the world [1]. The incidence rate is significantly increasing in a number of countries within Eastern Asia, which were historically at low risk but now most likely the result of increases by westernized lifestyle [2, 3]. Previous studies have shown that modifiable lifestyle-related factors, including low levels of physical activity and obesity, are associated with survival after diagnosis in CRC patients [4]. Gene expression changes which disrupt the mechanisms that normally control the growth of colonic epithelial cells have been investigated in CRC [5, 6]. However, the molecular mechanisms underlying the formation and development of CRC are still poorly understood.

Regenerating islet-derived 3 alpha (REG3A) is a member of REG protein family and also named as human hepatocarcinoma-intestine-pancreas (HIP) or human pancreatitis-associated protein (PAP) [7-9]. *REG3A* mRNA is expressed in pancreas and small intestine, while not evident in colon, kidney, lung or brain [7]. It is a secreted calcium-dependent lectin protein related with regeneration of pancreatic islet cells[10], activation of pancreatic stellate cells (PSCs) [11] and liver regeneration [12]. REG3A has been previously linked to a number of human cancers [13-16], including hepatocellular carcinoma, gastric carcinoma and CRC. A previous study demonstrated that REG3A treatment increases the deposition of collagen and fibronectin of PSCs [11], which suggested that it may be involved in cancer progression. During liver tumorigenesis, REG3A is a downstream target of the Wnt pathway [13]. However, no studies have yet demonstrated the exact role of REG3A on colorectal carcinogenesis and the details of the pathways through which signals REG3A exerted its function.

In this study, we showed that REG3A expression was significantly elevated in CRC tissues when compared with normal colon tissues. Further clinical characteristics analysis showed that higher expression level of REG3A was associated with bigger tumor size, poorer differentiation, higher tumor stage and lower survival rate. Functional assays and Genome Set Enrichment Analysis (GSEA) confirmed that REG3A might serve as an oncogene by regulating cell proliferation, apoptosis, migration and invasion through activating AKT and ERK pathways.

## RESULTS

### REG3A expression in human colorectal tissues

**Figure 1:**
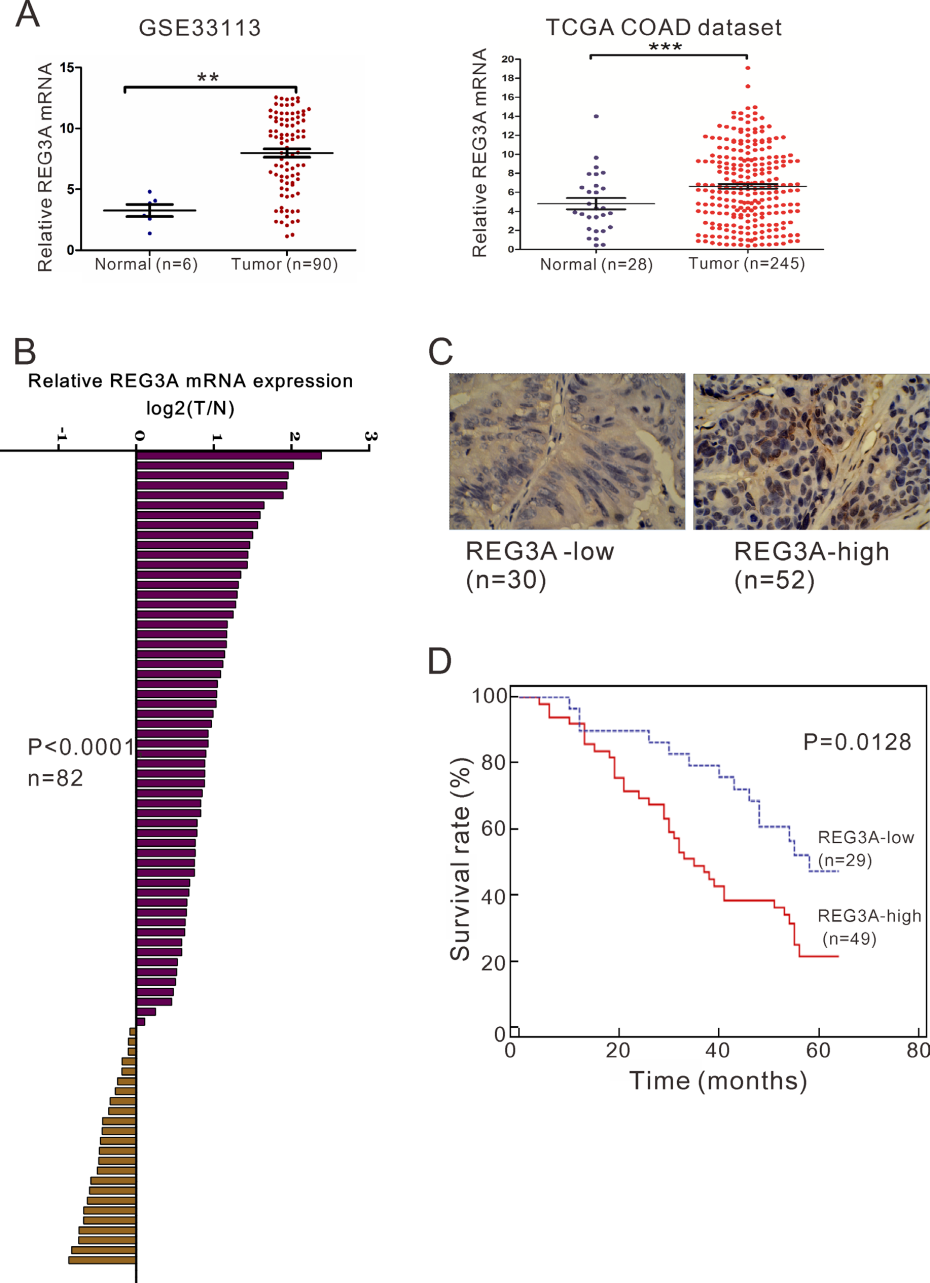
REG3A overexpression correlates with poor survival in CRC patients **A.** The expression level of REG3A in CRC and normal tissues based on two independent dataset: GSE33113 dataset (**P < 0.01) and TCGA COAD dataset (***P < 0.001). **B.** The mRNA levels of REG3A in 82 paired CRC and non-tumorous tissues were determined using qRT-PCR. **C.** Expression of REG3A was determined by immunohistochemistry staining in CRC tissues. Magnification: 200×. **D.** Survival analysis showed that REG3A-lower expression tumors have a favorable prognosis compared to REG3A-higher expression tumors (*P* = 0.0091, HR = 0.4176, 95% CI: 0.2373-0.7349).

To determine REG3A expression in CRC, we re-analyzed microarray data from ArrayExperss (Access id: GSE33113) and The Cancer Genome Atlas (TCGA) for the colon adenocarcinoma (COAD) dataset. We found that REG3A expression was significantly increased in CRC tissues when compared with the normal colon tissues (*P* < 0.01, *P* < 0.001, Figure 1A). Furthermore, quantitative RT-PCR analysis was performed on 82 pairs of primary CRC RNA samples. Clearly, REG3A was upregulated at RNA level in 70.7% (58/82) of tested CRC specimens (Figure 1B). To further verify this finding, we performed immunohistochemistry (IHC) staining. 30 and 52 cases of CRC tissues display high expression of REG3A (more than 20% of tumor cells were positively stained) and low expression of REG3A (less than 20% of tumor cells were positively stained), respectively (Figure 1C).

### Increased REG3A expression in CRC tissues correlated with patients’ survival

Further, according to the relative REG3A protein expression in tumor tissues, the 82 CRC patients were classified into two groups: relative high group (*n* = 52) and relative low group (*n* = 30). To explore the clinical significance of REG3A in CRC, we statistically analyzed the correlation between its expression levels and clinicopathologic features in the two groups of CRC patients based on the integrity of the clinical data. Interestingly, Fisher's exact test indicated that REG3A expression level was significantly correlated with tumor size (*P* = 0.0211), differentiation (*P* = 0.0213) and tumor stage (*P* = 0.0186), but not gender or age (Table 1). A multivariate Cox regression analysis confirmed REG3A expression, tumor size, and differentiation as independent predictors of the overall survival in CRC patients (Table 2).

**Table 1:** Correlation of REG3A expression in colon cancer tissues with different clinicopathological features (n = 82)

Characteristic	*n*	REG3A		*P*-value
		Low (*n* = 30)	High (*n* = 52)	
Gender				0.8153
Male	51	18	33	
Female	31	12	19	
Age (years)				0.8223
>=65	45	17	28	
<65	37	13	24	
Tumor size (cm)				0.0211*
<4.0	35	18	17	
>=4.0	47	12	35	
Differentiation				0.0213*
Well/Moderate	40	20	20	
Poor	42	10	32	
Stage				0.0186*
I/II	32	17	15	
III/IV	50	13	37	

Clinicopathological features were assessed using the Fisher's exact test.

* *P* < 0.05.

**Table 2:** Multivariate Cox regression of prognostic parameters for survival in patients with colon cancer

Prognostic parameter	Multivariate analysis		
	HR	95% CI	*P*-value
Expression of REG3A (low vs. high)	3.531	1.760-7.085	0.000***
Tumor size (< 4.0 cm vs. >= 4.0 cm)	2.827	1.526-5.238	0.001**
Differentiation (Well/Moderate vs. Poor)	0.216	0.113-0.414	0.000***
Stage (I/II vs. III/IV)	1.521	0.832-2.781	0.173

HR: Hazard ratio; CI: Confidence interval.

** *P* < 0.01

*** *P* < 0.01

We then investigated the correlation between REG3A protein expression and prognosis of CRC patients. Because operation for stage IV disease is usually in a palliative manner, four patients at Stage IV were excluded from overall survival analysis. Kaplan-Meier analysis showed that the overall survival time of REG3A-low-expressing group was notably longer than that of REG3A-high-expressing group (Figure 1D).

### Silencing of REG3A inhibited cell proliferation of CRC cells *in vitro* and *in vivo*

**Figure 2:**
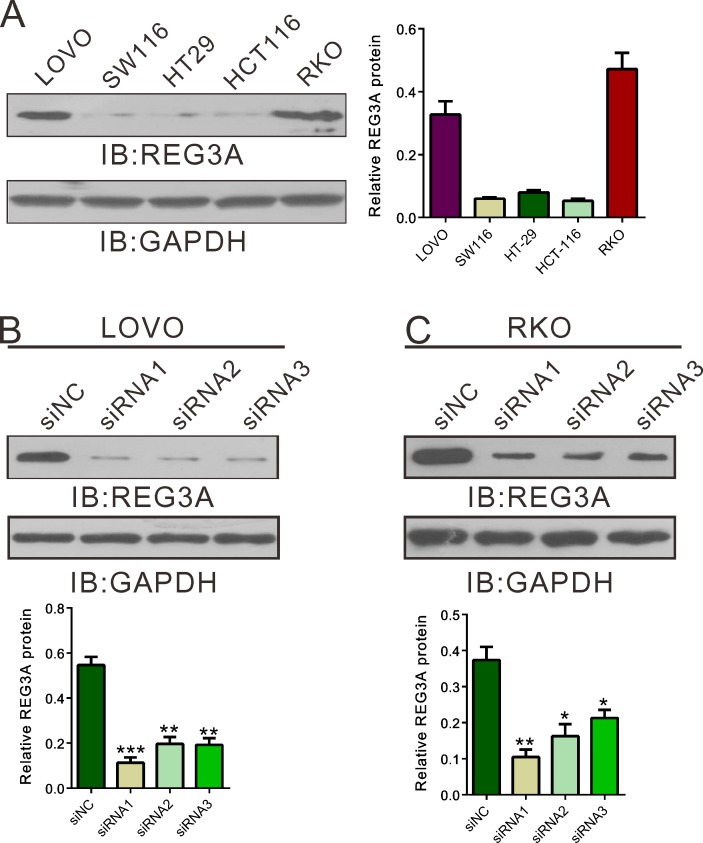
Suppressing of REG3A expression by siRNA transfection **A.** REG3A expression level in five CRC cell lines was analyzed by Western blot. **B.**, **C.** The efficiency of three siRNAs in knocking down endogenous REG3A in LOVO and RKO cells was evaluated using Western blot. Representative blot and quantification of western blot were shown. *P < 0.05, **P < 0.01, ***P < 0.001.

To explore the role of REG3A in CRC behavior, we transfected siRNAs targeting REG3A (siRNA1, siRNA2 and siRNA3) or control siRNA (siNC) into LOVO and RKO cells, which has relative higher REG3A level (Figure 2A). As shown in Figure 2B and 2C, all three siRNA efficiently down-regulated REG3A expression. siRNA1 and siRNA2 had higher knockdown efficiency than siRNA3, and chosen for further experiments.

**Figure 3:**
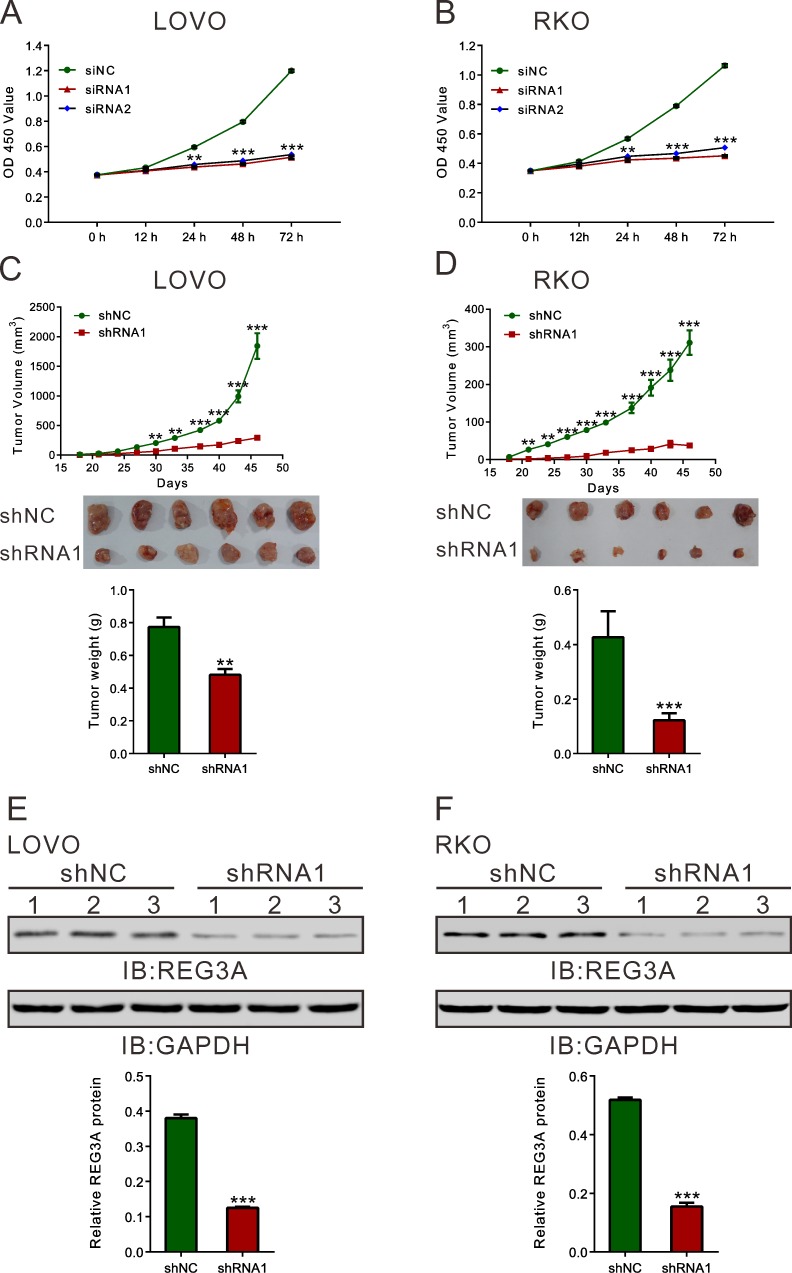
REG3A knockdown suppressed CRC cell proliferation *in vitro* and *in vivo* **A.**, **B.** Rate of cell proliferation was assessed by CCK-8 assay when LOVO and RKO cells were knocked down with REG3A siRNA1 and siRNA2. **C.**, **D.** Knockdown of REG3A significantly inhibited tumor growth in nude mice xenograft model. Tumor volume was measured for 46 days. At day 46, mice were sacrificed and tumors were weighted (*n* = 6). **E.**, **F.** The protein expression of REG3A in xenograft was assessed by Western blot. Representative blot and quantification of western blot were shown. **P < 0.01, ***P < 0.001.

We then used CCK-8 assay to measure the ability of cell proliferation (Figure 3A and 3B). Compared with siNC, both REG3A siRNA1 and siRNA2 significantly inhibited cell proliferation at 24, 48 and 72h after siRNA transfection. On the contrary, the proliferation of two lower-REG3A expression cells, HT-29 and SW116, was increased by REG3A overexpression (Figure S2A and S2B).

Next, we explored whether silence of REG3A in CRC cells could suppress tumor growth *in vivo*. REG3A short hairpin RNA lentivirus (shRNA1) and control lentivirus (shNC) were generated. LOVO or RKO cells stably transducted with shRNA1 or shNC were established and injected subcutaneously to nude mice. Tumor volumes were measured for 46 days. As shown in Figure 3C and 3D, the growth rates of REG3A silenced tumors were much slower compared with those of control tumors. After 46 days, the volume and weight of REG3A shRNA1 treated tumor was significantly smaller and lighter than that of control (*P* < 0.01). Meanwhile, REG3A protein expression decreased by 67.0% and 70.2% in xenograft formed by shRNA1-treated LOVO or RKO cells, respectively, as compared to xenograft formed by shNC-treated cells. Complementary results were observed in HT-29 and SW116 cells stably overexpressed REG3A (Figure S2C-F). These data suggested that inhibition of REG3A in CRC cells repressed cell proliferation *in vitro* and *in vivo*.

### Knockdown of REG3A induced G1 phase arrest and cell apoptosis in CRC cells

**Figure 4:**
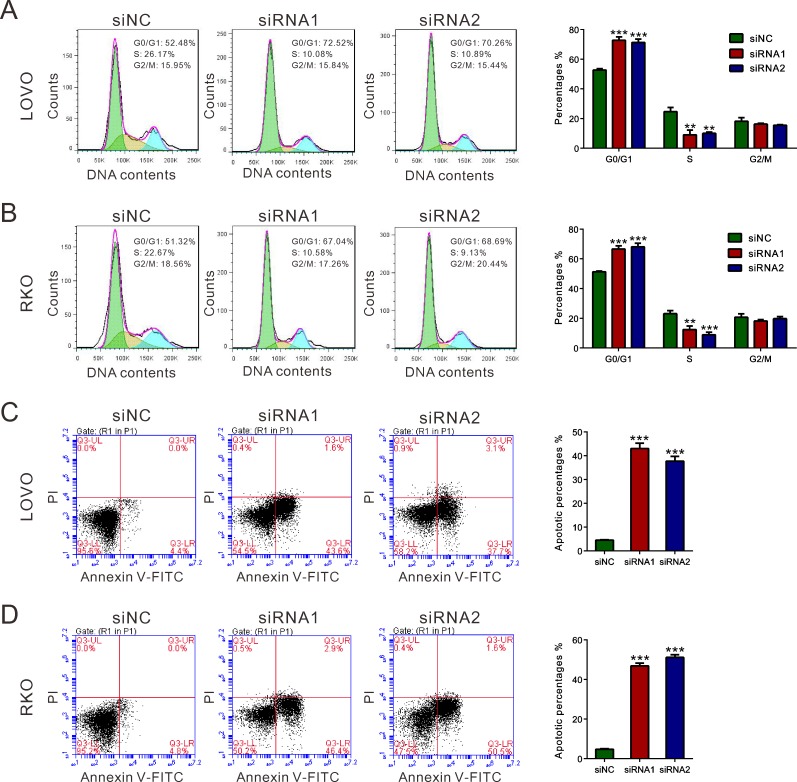
REG3A induced G1 phase arrest and cell apoptosis of CRC cells **A.**, **B.** The synthesized siRNAs against REG3A suppressed the G1-S phase transition. Cell cycle profile was analyzed using PI staining and flow cytometry. **C.**, **D.** Apoptotic cells were measured by Annexin V-PI staining when LOVO and RKO cells were knocked down with REG3A siRNAs. **P < 0.01, ***P < 0.001.

Because cell proliferation was reduced when REG3A was knockdown, we examined whether this inhibition is associated with cell cycle progression and cell apoptosis. Cell population at G0/G1 phase was markedly increased, while S-phase cells were notably decreased in CRC cells transfected with REG3A siRNA (Figure 4A and 4B). Transfection with REG3A siRNA1 and siRNA2 markedly increased the apoptotic ratio of cells compared to cells transfected with siNC (Figure 4C and 4D).

### Silencing of REG3A inhibited migration and invasive ability of CRC cells

**Figure 5:**
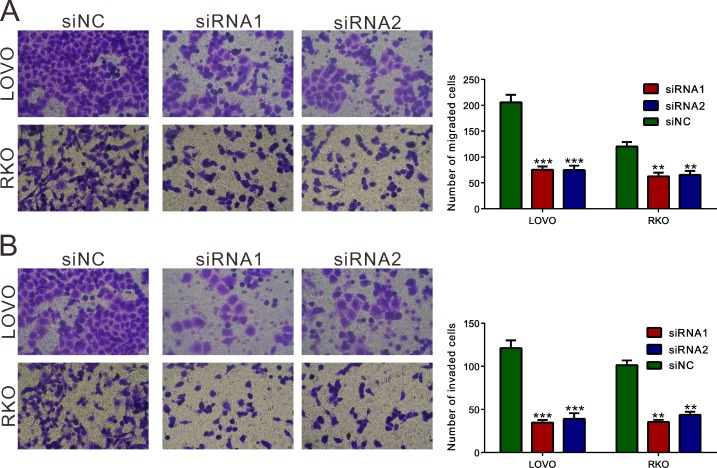
REG3A knockdown suppressed the cellular migration and invasion of CRC cells Migration **A.** and invasion **B.** assay were performed in Transwell chambers. For invasion assay, the upper chamber was pre-coated with Matrigel. **P < 0.01, ***P < 0.001.

To determine whether REG3A influences migration and invasion of CRC cells, transwell assay was performed and the number of migrated and invaded cells was assessed following a 24h-culture period. Cells transfected with REG3A siRNA1 and siRNA2 showed a significant reduction in cell migration and invasion compared to control siNC-transfection cells (Figure 5A and 5B). Complementary to the results from RNAi, REG3A overexpression significantly increased the migration and invasion of HT-29 and SW116 cells (Figure S3).

### REG3A-associated pathways in CRC

**Figure 6:**
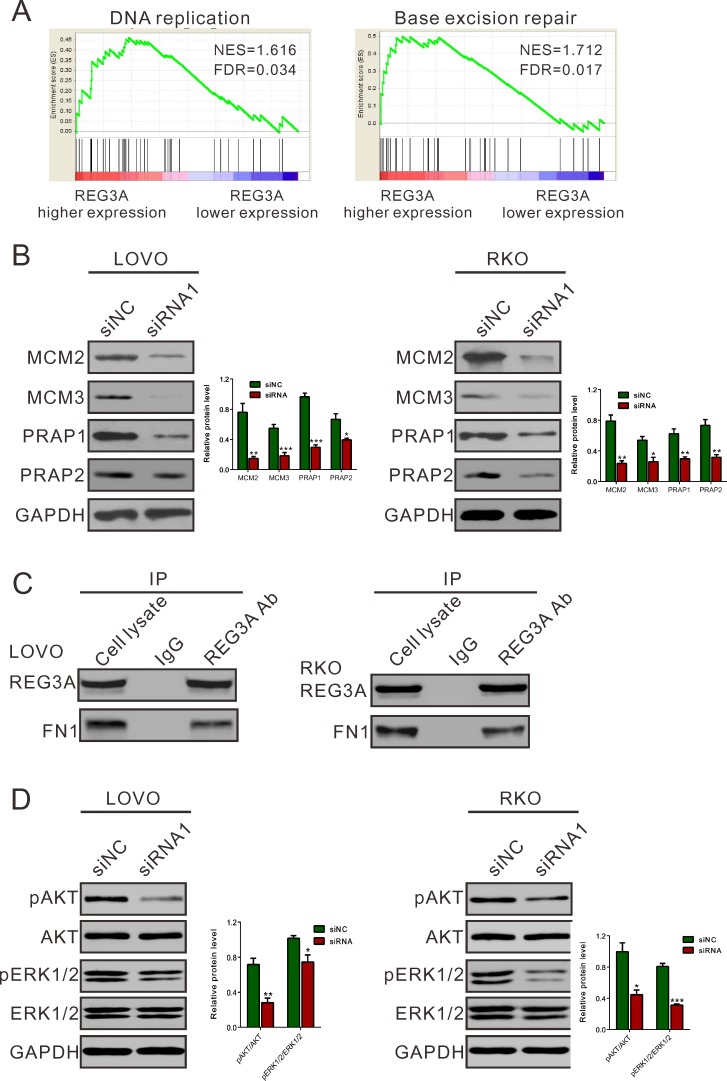
Mechanisms how REG3A exerts its function in CRC **A.**, **B.** GSEA analysis in CRC patients with higher REG3A expression versus lower REG3A expression based on TCGA CORD datasets. NES, normalized enrichment score. DNA replication and base excision repair (BER) pathways have the strongest association with REG3A-higher expression. **C.** Protein levels of key proteins in DNA replication and BER pathways were determined by Western blot. **D.** Co-immunoprecipitation analysis showed that REG3A interacts with fibronectin1 (FN1). **E.** REG3A siRNA suppressed activation of AKT and ERK1/2 pathway. *P < 0.05, **P < 0.01, ***P < 0.001.

To further explore the role of REG3A in CRC, we performed Gene Set Enrichment Analysis (GSEA) in CRC samples with higher REG3A expression versus lower REG3A expression based on TCGA CORD dataset. Our data implyed that higher REG3A expression was positively correlated with Kyoto Encyclopedia of Genes and Genomes (KEGG) DNA replication and base excision repair (BER) pathways in CRC samples (Figure 6A).

To further validate the GSEA results, we then detected the protein expression of DNA replication (MCM2 and MCM3) and BER pathways-related proteins (PARP1 and PARP2) in REG3A silenced CRC cells. The levels of detected protein were significantly decreased in both LOVO and RKO cells (Figure 6B) after the downregulation of REG3A.

Fibronectin1 (FN1) was identified as a potential interaction partner for REG3A in rat hepatocyte [9]. The growth-promoting effect of FN1 has been explored in non-small cell lung carcinoma cell [17]. We then performed co-immunoprecipitation (IP) experiments in CRC cells using REG3A antibody. The results indicated that the endogenous REG3A and FN1 were immunoprecipitated by REG3A antibody, but not by the control IgG (Figure 6C). These data suggested that REG3A might exert its function in CRC through interacting with FN1.

Phosphatidylinositol 3 kinase (PI3K)-AKT [18, 19] and ERK1/2 [20] pathways is frequently activated in human cancer, exerting growth-promoting, metastasis-promoting and antiapoptotic activities in tumor cells. Lai et al. [21] found that REG3A increases keratinocyte proliferation by promoting AKT pathway. Kadowaki et al. [22] reported that REG activated ERK1/2, which would strongly stimulate the proliferation of gastric cancer cells. We then examine the effect of REG3A on AKT and ERK1/2 activity in CRC cells. REG3A siRNA1 transfection significantly reduced the phosphorylation of AKT and ERK1/2 in LOVO and RKO cells (Figure 6D). Complementary to the results from RNAi, ectopic expression of REG3A in HT-29 and SW116 cells notably increased activity of AKT and ERK1/2 (Figure S4).

## DISCUSSION

REG3A belongs to REG protein family. Members of REG protein family, such as REG1, REG3A and REG4, have been found to be up-regulated in human CRC tissues [16, 23-26]. However, the previous studies did not clarify the roles of REG3A in CRC behaviors. In this study, we identified REG3A as a potential oncogene in CRC based on analysis of public datasets and clinical subjects, and *in vitro* and *in vivo* functional experiments.

Firstly, our results demonstrated the overexpression of REG3A in CRC tissues (Figure 1). Then we revealed that REG3A expression levels were significantly correlated with tumor size, differentiation and grade (Table 1), suggesting that REG3A overexpression is positively correlated with CRC progression. More importantly, multivariate analysis indicated that REG3A expression was an independent risk factor for poor prognosis of CRC patients (Table 2). Worse survival rate with higher expression of REG3A was observed in CRC patients (Figure 1D). Thus, we reported the potential clinical value of REG3A in patients with CRC.

Then we examined the functions of REG3A in CRC cells. a set of experiments revealed that silencing of REG3A in CRC cells with higher expression of REG3A (LOVO and RKO cells) could suppress cellular proliferation (Figure 3A and 3B), cellular migration, invasion (Figure 5) and *in vivo* tumorigenicity (Figure 3C and 3D). On the contrary, ectopic expression of REG3A in in CRC cells with lower expression of REG3A (HT-29 and SW116 cells) cause an inverse effect were observed in CRC cells (Figure S1 and S2). These data suggested for the first time that REG3A may be a potential oncogene in CRC.

In this work, in addition to cellular phenotype, we explore the molecular mechanism by which REG3A functions as an oncogene in CRC. Normal cellular division requires proper and timely control of DNA replication, while uncontrolled DNA replication could lead to aberrant cell proliferation and cancer development [27]. Increased expression or activity of genes involved in DNA repair [28] may protect cancer cells from cell apoptosis and enhance cell viability. In the present study, GSEA data indicated that higher REG3A expression was positively correlated with KEGG DNA replication and base excision repair (BER) pathways in CRC samples (Figure 6A). The expression of DNA replication (MCM2 and MCM3 [29]) and BER pathway (PARP1 and PARP2 [30]) related factors was significantly suppressed by REG3A knockdown. These data suggested the roles of REG3A on DNA replication and DNA repair, accounting for CRC carcinogenesis.

Further, REG3A was previously identified as a secreted protein and induced by interleukin-17 (IL-17), thus stimulating the proliferation and inhibit terminal differentiation of keratinocyte during skin injury through phosphatidylinositol 3 kinase (PI3K)/AKT pathway [21]. A previous study proved that ERK1/2 pathway lies downstream of REG signaling [22]. Fibronectin1 (FN1) is identified as a potential interaction partner for REG3A [9]. The previous studies revealed that FN1 could stimulated the growth of non-small cell lung carcinoma cell [17] *via* activating AKT signaling, and stimulated lung carcinoma cell growth *via* the phosphorylation of ERK [31]. Here, we demonstrated the interaction of REG3A and FN1 in CRC cells (Figure 6C) and the inhibitory effects of REG3A siRNA on the phosphorylation of AKT and ERK1/2 (Figure 6D). Our data suggested that REG3A may perform its biological function through AKT and ERK1/2 pathways. However, whether the interaction of REG3A and FN1 enhanced the activation of AKT or ERK1/2 requires further investigation.

In conclusion, our study suggest that REG3A expression play an important role in the extensive network of heterogeneous cellular pathways underlying colorectal tumorigenesis. Our data provided a deeper insight into the molecular mechanisms for the association between REG3A expression and the risk of CRC.

## MATERIALS AND METHODS

### Study subjects

CRC tissues and adjacent non-tumorous liver tissue counterparts used for qRT-PCR and Western blot were collected from 82 CRC patients who underwent surgical treatment between Jan 2007 and Dec 2008 at Shanghai Seventh People's Hospital (Shanghai, China). Tissue samples were snap frozen in liquid nitrogen immediately after surgical resection and stored at −80°C. Tumor stage was determined according to the TNM classification. This study did not include the patients who had received radiotherapy and/or immunotherapy before or after surgical treatment. Clinical information, such as gender, age, tumor size, tumor differentiation and tumor stage were obtained by review of medical records of the patients. The mean age of the patients was 64 years (ranging from 33 to 79 years), 65.8 % of whom are men. The study protocol conformed to the ethical guidelines of the 1975 Declaration of Helsinki, and was approved by the Institutional Ethical Review Committee of Shanghai Seventh People's Hospital (IE06012). All patients enrolled in the study gave written informed consents.

### Immunohistochemistry (IHC) analysis

After deparaffinized in xylene and rehydration in ethanol, the sections were antigen-retrieved by microwave treatment in 0.01M citrate buffer (pH 6.0) for 15 min. The sections were treated with 0.3% hydrogen peroxide for 30 min to inactive endogenous peroxidases, and then incubated with 10% normal goat serum for 30 min to block non-specific antigens. After incubation with REG3A antibody (Abcam, Cambridge, MA, USA) overnight at 4°C, the slides were incubated with horseradish peroxidase (HRP) conjugated secondary antibody for 1 h at room temperature. Immunoreactivity was detected with the 3,3-diaminobenzidine (DAB) solution (Vector Laboratories, Burlingame, CA, USA) and counterstained slightly with hematoxylin. The specimens were graded into REG3A-high-expression group (>20% of tumor cells showed positive stain) and REG3A-low-expression group ( < 20% of the tumor cells showed positive stain).

### Cell culture

HEK293T and human colorectal carcinoma cell lines, LOVO, SW116, HCT116, HT29 and RKO were obtained from the Shanghai Cell Bank, Chinese Academy of Sciences (Shanghai, China). Cells were cultured in DMEM supplemented with 10% fetal bovine serum (FBS), 100 units/mL penicillin and 100 μg/mL streptomycin, and incubated in a humidified atmosphere at 37°C with 5% CO_2_.

### RNA interference

Three siRNA targeting human REG3A mRNA (siRNA1: 5′-GUCUCAGGUUCAAGGUGAAUU-3′, siRNA2: 5′-GCAGUGAUGUGAUGAAUUAUU-3′ and siRNA3: 5′-CUGACCACCUCAUUCUUAUUU-3′) and a non-specific scramble siRNA sequence (NC) were synthesized, transiently transfected into LOVO and RKO cells using Lipofectamine 2000 (Invitrogen, Carlsbad, CA, USA) according to the manufacture's instruction. Assays were performed 48 h after transfection.

### RNA extraction and quantitative RT-PCR

Total RNA was extracted from the patients’ tissues samples using Trizol (Invitrogen Carlsbad, CA, USA) and treated with DNase I (Roche, Indianapolis, IN, USA) to remove residual DNA according to the manufacturer's protocol. qRT-PCR was used to examine the expression levels of REG3A mRNA. A total of 2 μg RNA was reversely transcribed to cDNA with oligo(dT) primer using cDNA synthesis kit (Thermo Fisher, Rockford, IL, USA), and then used for quantitative PCR with SYBR Green qPCR Master Mixes (Thermo Fisher) according to manufacturer's instruction. GAPDH mRNA levels were used for normalization. The oligonucleotides used as PCR primers were: *REG3A* 5′-TAATGTGAGGTTACCCTATG-3′ and 5′-GAGGAAGAAACAGAAGAAAG-3′; GAPDH 5′-CACCCACTCCTCCACCTTTG-3′ and 5′-CCACCACCCTGTTGCTGTAG-3′. ABI 7300 system (Applied Biosystem, Foster City, CA, USA) was programmed to initially incubate the samples at 95°C for 10 min, and then denature at 95°C for 10 min, followed by 40 cycles of 95°C for 15 s and 60°C for 45 s. *REG3A* mRNA expression was calculated using the ΔΔ Ct method. All data represent the average of three replicates.

### Coimmunoprecipitation (co-IP) assays

Cell lysates were reacted with Anti-REG3A (Ab134309, Abcam) or control IgG (Abcam) for 1 h at 4°C, then with protein A-agarose (150 μg protein A) for 3 h at 4°C. Precipitates were washed three times in lysis buffer and detected by western blot analysis.

### Western blot analysis

For tissue samples, about 0.2 g of tissue was grinded into powder in liquid nitrogen. Protein extracts of frozen tissue powder and cells were prepared by using RIPA buffer in the presence of proteinase inhibitor cocktail (Sigma, St. Louis, MO, USA). Total protein extracts were separated by SDS-PAGE and transferred onto nitrocellulose membranes (Millipore, Bredford, USA). Anti-REG3A (Abcam, Ab134309), anti-MCM2 (Abcam, Ab4461), anti-MCM3 (Abcam, Ab128923), anti-PRAP1 (Abcam, Ab118186), anti-PRAP2 (Abcam, Ab176330), anti-pAKT(Cell Signaling Technology (CST), #4060, Danvers, MA, USA), anti-AKT (CST, #4691), anti-pERK1/2 (CST, #9101S), anti-ERK1/2 (CST, #9102S) and anti-GAPDH (CST, #5174) were used in Western analysis in accordance with the manufacturer's instruction. Signals were visualized with enhanced chemiluminescenct substrate (ECL, BioRad, Richmond, CA, USA) by exposure to films.

### Cell proliferation assay

The viability of RKO and LOVO cells post-transfection was determined using Cell Counting Kit-8 (Dojindo Laboratories, Japan). Cells were seeded in 96-well plates at a density of 2 × 10^3^ cells per well and transfected with the desired siRNA followed by incubation for 12, 24, 48 and 72 h. At the end of incubation, 10 μl CCK-8 reagents was added to each well and incubated at 37°C for another 1 h. The number of viable cells was assessed by measurement of absorbance at 450 nm using Multiskan MS plate reader (Labsystems, Helsinki, Finland).

### Cell cycle analysis

Cells were harvested at 48 h after siRNA transfection. The cells were washed with PBS and fixed in ethanol at −20°C. The cells were then washed with PBS, rehydrated and resuspended in propidium iodide (PI)-RNase A solution (Sigma) at 37°C for 30 min. The stained cells (1 × 10^5^) were then analyzed for DNA content with a flow cytometer (BD Biosciences, Franklin Lakes, NJ, USA).

### Evaluation of apoptosis by flow cytometry

Cells were harvested, washed with ice-cold PBS, and stained with annexin V-fluorescein isothiocyanate (FITC) apoptosis detection kits (KeyGEN Biotech, Nanjing, China). Cell apoptosis was analyzed in a flow cytometer (BD Biosciences).

### Cell migration and invasion assay

Migration of cells was assayed using chamber with 8 μm pore filters (Corning, New York, NY, USA). Cells were grown to about 50% confluency and transfected with the desired siRNA. After 24 h, the cells were incubated in serum-free medium for 24 h. Then cells were trypsinized and 5 × 10^4^ cells in serum-free medium added to the upper chamber. Then media with 10% FBS was added to the lower chamber. Cells were incubated for 24 h at 37°C, and then non-migrating cells were completely removed. Cells that migrated to the bottom of the membrane were then fixed in 4% paraformaldehyde and stained by 0.5% crystal violet. Then stained cells were visualized under a microscope, counted in five random fields, and the average number was taken.

For invasion assay, the upper chamber was pre-coated with 1 mg/ml Matrigel (BD Biosciences). The rest of the assay was performed as migration assay.

### Establishment of Stable cell lines and *In vivo* tumorigenicity assay

shRNA targeting human REG3A mRNA (GTCTCAGGTTCAAGGTGAATT, shRNA1) was cloned into a lentiviral vector (PLKO.1, Addgene, Cambridge, MA, USA). A non-specific scramble shRNA sequence (shNC) was used as negative control. Lentivirus was produced by transfecting lentiviral vector and lentiviral packaging vectors into HEK293T cells by using lipofectamine 2000 (Invitrogen). At 48 h after transfection, viruses were collected to transduct LOVO and RKO cells. Stable cell line was established by puromycin (Sigma) selection.

The animal study protocol was approved by the Animal Experimentation Ethics Committee of Shanghai Seventh People's Hospital. Twenty-four BALB/c nude mice aged 4-5 weeks old (SLAC Animal, Shanghai, China) were housed in aseptic conditions and maintained under constant humidity and temperature. LOVO and RKO cells stably transducted with REG3A shRNA1 or shNC were harvested and injected subcutaneously into each of the six nude mice (2×10^6^). Tumor volume was monitored every three days by calculating using the following formula: volume = 1/2 × (largest diameter) × (smallest diameter)^2^. 46 days later, the mice were sacrificed, the tumors were recovered and the wet weights of each tumor were examined.

### Bioinformatics analysis

The gene expression data were obtained at The Cancer Genome Atlas website (TCGA, https://tcga-data.nci.nih.gov/tcga/) for the colon adenocarcinoma (COAD) projects or GEO dataset (Access id: GSE33113, http://www.ncbi.nlm.nih.gov/geo/query/acc.cgi?acc=GSE33113).

Our Gene set enrichment analysis (GSEA) of pathways and genes was performed based on TCGA CORD dataset using the GSEA version 2.0 from the Broad Institute at MIT. In our analysis, the gene sets of fewer than 10 genes were excluded. The t-statistic mean of the genes was computed in each KEGG (Kyoto Encyclopedia of Genes and Genomes) pathway. Using a permutation test 1,000 times, the cutoff of the significance level p values was chosen as 0.01 for the most significant pathways related to REG3A expression.

### Statistical analysis

Clinicopathological features, such as gender, age, tumor size, differentiation and tumor stage, were assessed using the Fisher's exact test. Kaplan-Meier survival plots were generated for patients with low or high tumor REG3A expression, and the significance was determined by log-rank analysis. Student's t test was also used to evaluate the expression values *in vitro* functional and animal experiment. All statistical analyses were two-sided and done using the Statistical Package for the Social Sciences software version 16.0 (SPSS, Inc., Chicago, IL, USA). Statistical significance was set at *P* < 0.05.

## SUPPLEMENTARY MATERIAL FIGURES


