# Supplementary material for: Up-regulation of REG3A in colorectal cancer cells confers proliferation and correlates with colorectal cancer risk
Source: Oncotarget. 2015 Dec 4;7(4):3921–33. doi: 10.18632/oncotarget.6473 (PMC4826180; doi:10.18632/oncotarget.6473)
Supplement: Supplementary file 1 [file oncotarget-07-3921-s001.pdf]

Up-regulation of REG3A in colorectal cancer cells confers proliferation and correlates with colorectal cancer risk

Supplementary Material

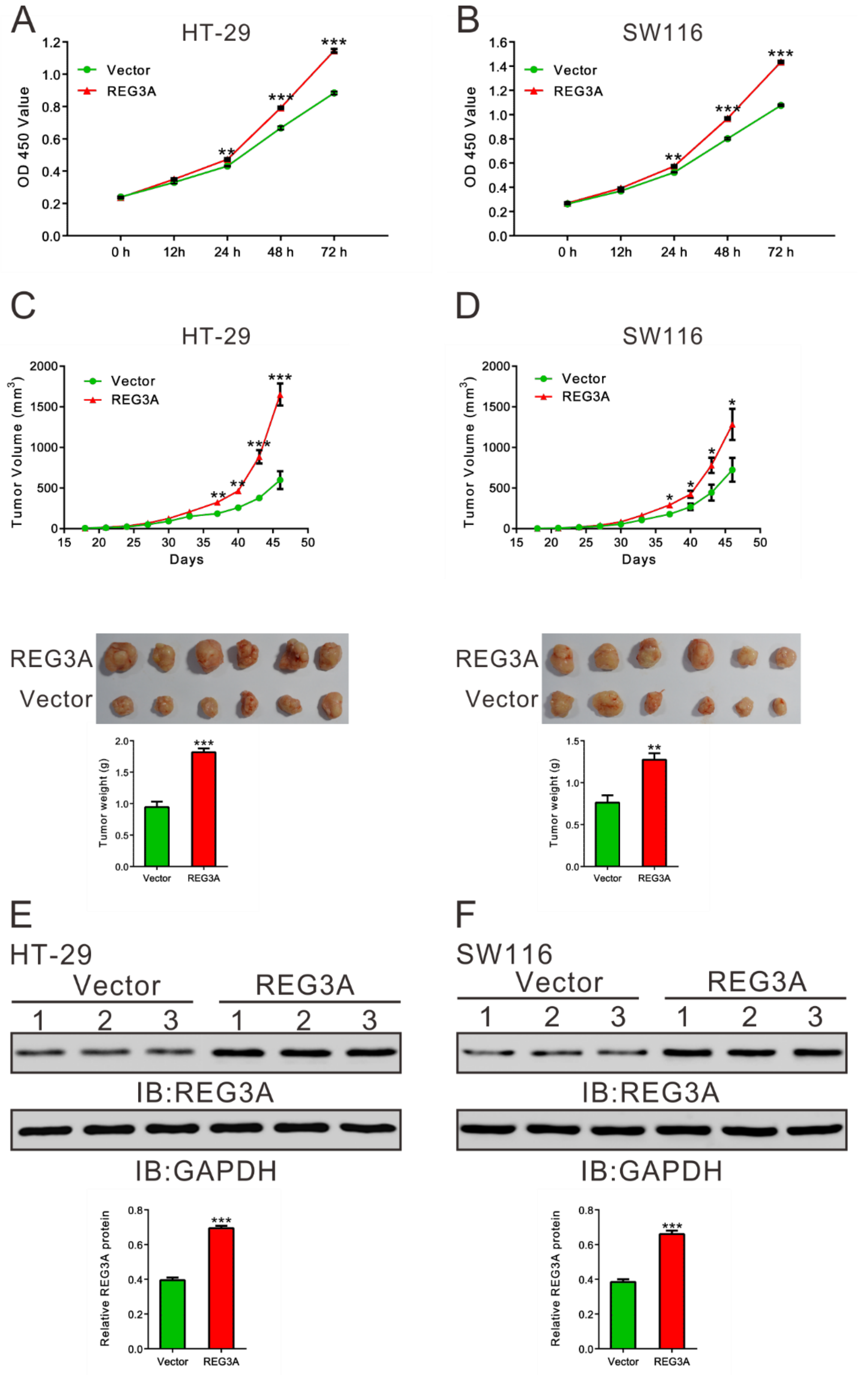

Figure S1. Effects of REG3A overexpression on cell proliferation in vivo and in vitro.

(A, B) The full-length REG3A were cloned into the expression vector pLVX-AcGFP1-C1 (Clontech, Palo Alto, CA, USA). REG3A expressing lentivirus (REG3A) and control lentivirus (Vector) were generated. HT-29 and SW116 cells were transducted with lentivirus and cell proliferation was assessed by CCK-8 assay at 0, 12h, 24h, 48h and 72h post virus treatment. (C, D). HT-29 and SW116 cells stably expressed REG3A were generated, harvested and injected subcutaneously into nude mice. Forced expression of REG3A significantly inhibited tumor growth in nude mice xenograft model (n=6). (E, F) REG3A protein expression in xenograft was assessed by Western blot. Representative blot and quantification of western blot were shown. ***P*<0.01, ****P*<0.001.

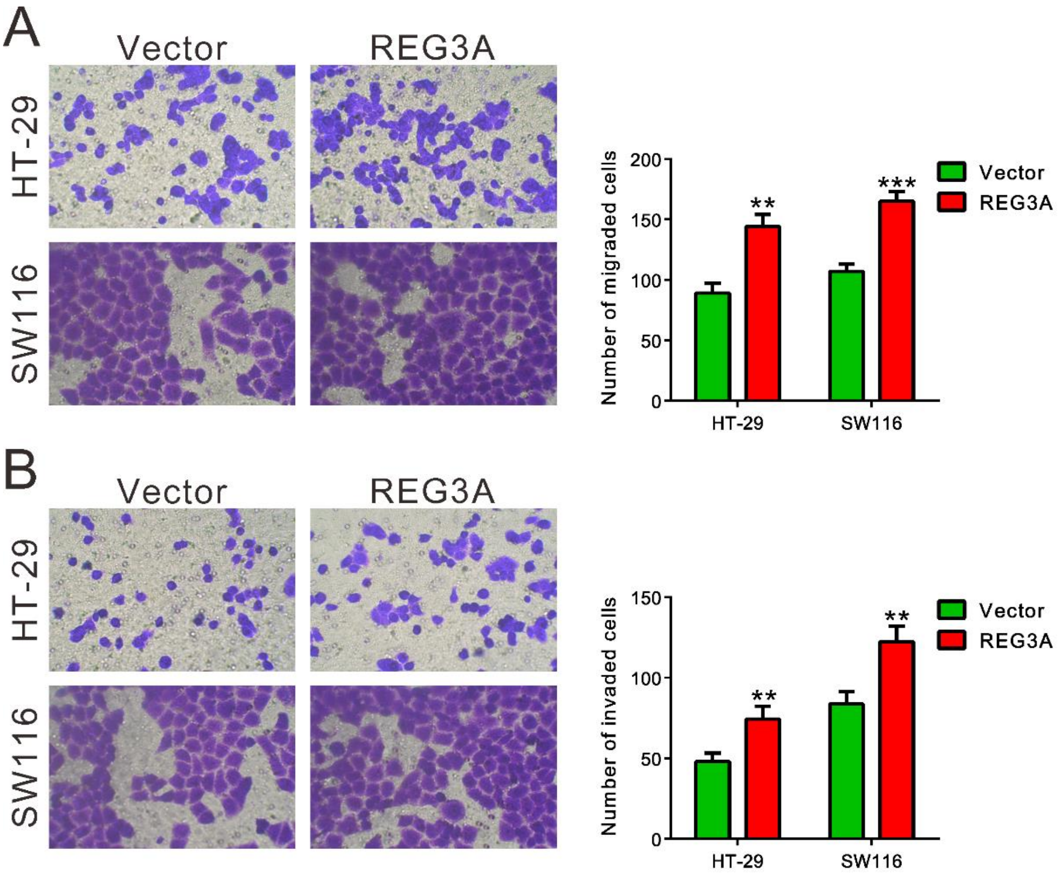

Figure S2. Effects of REG3A overexpression on cell migration and invasion. Migration (A) and Invasion (B) assay were performed in Transwell chambers. For invasion assay, the upper chamber was pre-coated with Matrigel. $^{**}P<0.01$, $^{***}P<0.001$.

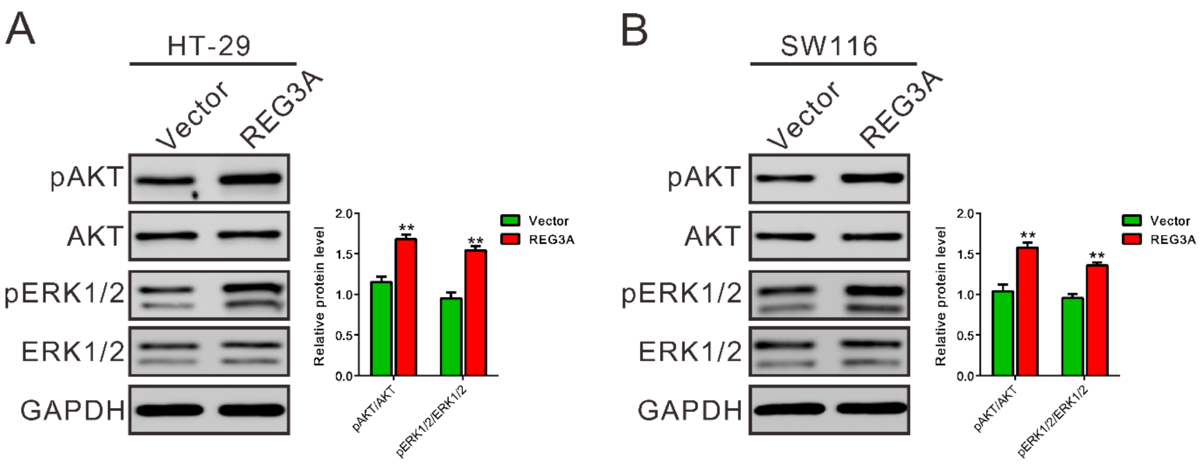

Figure S3. Activity of AKT and ERK signal pathways was determined by western blotting. Data were presented as the mean value from three independent experiments ± S.D. ***P*<0.01.
